# Covered TIPS for secondary prophylaxis of variceal bleeding in liver cirrhosis: A systematic review and meta-analysis of randomized controlled trials

**DOI:** 10.1097/MD.0000000000005680

**Published:** 2016-12-16

**Authors:** Xingshun Qi, Yulong Tian, Wei Zhang, Haitao Zhao, Guohong Han, Xiaozhong Guo

**Affiliations:** a Liver Cirrhosis Study Group & Meta-analysis Study Interest Group, Department of Gastroenterology, General Hospital of Shenyang Military Area; b Department of Interventional Radiology & Key Laboratory of Diagnostic Imaging and Interventional Radiology of Liaoning Province, First Affiliated Hospital of China Medical University; c Medical Ethical Committee, General Hospital of Shenyang Military Area, Shenyang; d Xijing Hospital of Digestive Diseases, Fourth Military Medical University, Xi’an, China.

**Keywords:** endoscopy, liver cirrhosis, portal hypertension, prevention, propranolol

## Abstract

**Background:**

In the era of bare stents, transjugular intrahepatic portosystemic shunt (TIPS) is the second-line choice of therapy for the prevention of variceal rebleeding in liver cirrhosis. In the era of covered stents, the role of TIPS should be re-evaluated.

**Aim:**

The aim of the study was to compare the outcomes of covered TIPS versus the traditional first-line therapy (i.e, drug plus endoscopic therapy) for the prevention of variceal rebleeding in liver cirrhosis.

**Methods:**

All relevant randomized controlled trials were searched via the PubMed, EMBASE, and Cochrane Library databases. Hazard ratios (HRs) and odds ratios (ORs) with 95% confidence intervals (CIs) and *P* values were calculated for the cumulative risk and overall risk, respectively. Heterogeneity among studies was also calculated.

**Results:**

Three of 111 retrieved papers were eligible. Among them, the proportion of patients who were switched from drug plus endoscopic therapy to TIPS was 16% to 25%. The risk of bias was relatively low in all included randomized controlled trials. Meta-analyses demonstrated that the covered TIPS group had a similar overall survival (HR = 0.84, 95% CI = 0.55–1.28, *P* = 0.41; OR = 1.00, 95% CI = 0.59–1.69, *P* = 0.99), a significantly lower risk of variceal rebleeding (HR = 0.30, 95% CI = 0.18–0.48, *P* < 0.00001; OR = 0.24, 95% CI = 0.12–0.46, *P* < 0.0001), and a similar risk of hepatic encephalopathy (HR = 1.35, 95% CI = 0.72–2.53, *P* = 0.36; OR = 1.28, 95% CI = 0.54–3.04, *P* = 0.57). In most of meta-analyses, the heterogeneity among studies was not statistically significant.

**Conclusions:**

Compared with drug plus endoscopic therapy, covered TIPS had a significant benefit of preventing from variceal rebleeding, but did not increase the overall survival or risk of hepatic encephalopathy.

## Abstract 2

Supplemental Digital Content is available in the text

## 1 Introduction

The incidence of variceal rebleeding is about 60% per year in survivors after an episode of variceal bleeding.^[1–3]^ Prevention strategy of variceal rebleeding should be actively initiated after the first episode of variceal bleeding.^[1–3]^ According to the current practice guideline recommendation, endoscopic variceal ligation in combination with nonselective beta-blockers should be the first-line choice of therapy for the prevention of variceal rebleeding in liver cirrhosis and that transjugular intrahepatic portosystemic shunt (TIPS) is the second-line choice of therapy.^[2]^ This is primarily based on the results of previous meta-analyses of randomized controlled trials that TIPS decreases the incidence variceal rebleeding, but increases the incidence of hepatic encephalopathy, carries a high incidence of shunt dysfunction, and does not improve the overall survival.^[2,4–6]^ Notably, all randomized controlled trials included in the previous meta-analyses were performed with bare stents for TIPS procedures. Emerging evidence suggested the benefits of covered stents for TIPS procedures in terms of shunt patency and hepatic encephalopathy.^[7–11]^ Therefore, it should be necessary to update the role of TIPS for the prevention of variceal rebleeding in the era of covered stents.

## 2 Methods

### 2.1 Study registration

This meta-analysis was registered on PROSPERO (No. CRD42016038200). The ethical approval or informed patient consent was not necessary, because this work was a systematic review and meta-analysis of randomized controlled trials, but not a clinical study in humans.

### 2.2 Search strategy

We searched 3 major databases, including the PubMed, EMBASE, and Cochrane Library databases on April 17, 2016. The search items were “(Covered stent) OR (Fluency) OR (Viatorr)” AND “(transjugular intrahepatic portosystemic shunt) OR (TIPS)” AND “randomized.”

### 2.3 Eligibility criteria

We identified all randomized controlled trials which compared the outcomes of covered TIPS versus drug plus endoscopic treatment for the prevention of variceal rebleeding. In details, according to the PICOS rule, the participants should be cirrhotic patients with previous variceal bleeding, the interventional group should be cirrhotic patients who underwent TIPS with covered stents, the control group should be cirrhotic patients who underwent the traditional first-line treatment option for the secondary prophylaxis of variceal bleeding (e.g., drug plus endoscopic treatment), the outcomes should be overall survival, variceal rebleeding, and/or hepatic encephalopathy, and the study design should be randomized controlled trials. Exclusion criteria were as follows: (1) duplicates; (2) narrative or systematic reviews; (3) protocols; (4) case reports; (5) nonrandomized studies; (6) TIPS with covered stents was not the interventional group; and (7) no comparison between covered TIPS versus drug plus endoscopic treatment for the secondary prophylaxis of variceal bleeding.

### 2.4 Data extraction

We extracted the following data: journal, publication year, region, enrollment period, major characteristics of study population, Child–Pugh class, follow-up duration, number of patients randomized, mortality, rate of variceal rebleeding, and rate of hepatic encephalopathy.

### 2.5 Risk of bias

We employed the revised “risk of bias” tool described in Cochrane Handbook version 5.1.0 to evaluate the study quality. It included 5 major domains (i.e., selection bias, performance bias, detection bias, attrition bias, and reporting bias) with 6 questions (i.e., random sequence generation, allocation concealment, blinding of participants and personnel, blinding of outcome assessment, incomplete outcome data, and selective reporting). The judgment for every question should be expressed as “low risk,” “high risk,” or “unclear risk” of bias.

### 2.6 Data analysis

We performed the meta-analyses by using random-effect models in the Review Manager 5.3. Forest plots were drawn. As we previously mentioned,^[12–15]^ hazard ratios (HRs) and odds ratios (ORs) with 95% confidence intervals (CIs) and *P* values were calculated as the effect sizes for cumulative risk and overall risk, respectively. In details, *P* < 0.05 was of statistically significant difference. *I*^2^ and *P* values were calculated to evaluate the heterogeneity among studies. In details, *I*^2^ > 50% and/or *P* < 0.1 were of statistically significant heterogeneity. Otherwise, the heterogeneity was not statistically significant. Due to a small number of included studies, the sensitivity or subgroup analyses were not performed, and the funnel plots were not drawn.

## 3 Results

### 3.1 Study selection and characteristics

**Figure 1:**
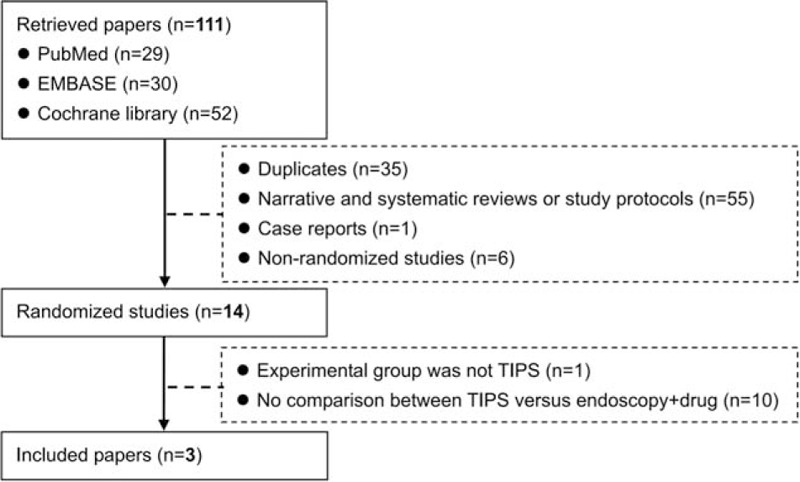
Flowchart of study inclusion.

Among the 111 retrieved studies, 3 randomized controlled trials were included in our study^[16–18]^ (Fig. 1). Study characteristics were shown in Table 1. The major study characteristics were summarized as follows.

(1) They were conducted between 2006 and 2013 and published after 2015.
(2) Two randomized controlled trials by Holster and Sauerbruch were conducted in European multicenters, and another one randomized controlled trial by Luo was conducted in a Chinese single center.
(3) In 1 randomized controlled trial by Luo, only cirrhotic patients with portal vein thrombosis were included; in 1 randomized controlled trial by Sauerbruch, patients with pre-hepatic portal hypertension were excluded; and in the remaining 1 randomized controlled trial by Holster, patients with portal hypertension resulting from other causes than liver disease (e.g., portal or splenic vein thrombosis) were excluded.
(4) In 2 randomized controlled trials by Holster and Sauerbruch, the proportion of Child–Pugh class A was 36.1% and 47.0%, respectively, and in another one randomized controlled trial by Luo, no patient had Child–Pugh class A.
(5) As for the experimental group, in 2 randomized controlled trials by Holster and Sauerbruch, Viatorr covered stents were employed for TIPS procedures; and in another one randomized controlled trial by Luo, Fluency covered stents were employed for TIPS procedures.
(6) As for the control group, in 2 randomized controlled trials by Holster and Luo, variceal band ligation plus nonselective beta-blockers were employed; and in another one randomized controlled trial by Sauerbruch, the hepatic venous pressure gradient (HVPG)-guided therapeutic strategy (e.g., the HVPG responders received only nonselective beta-blockers and nitrate, but the nonresponders were switched to TIPS) was employed.
(7) As for the control group, the proportion of patients who were switched to TIPS was 16% to 25%.

**Table 1:**
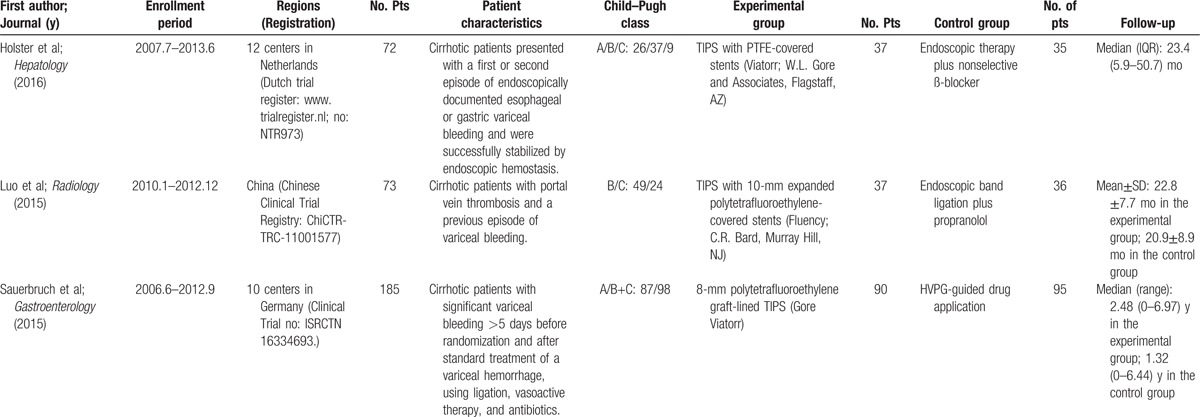
Study characteristics.

### 3.2 Risk of bias

Risk of bias for every individual randomized controlled trial was summarized in Supplementary Tables 1–3.

### 3.3 Overall survival

**Figure 2:**
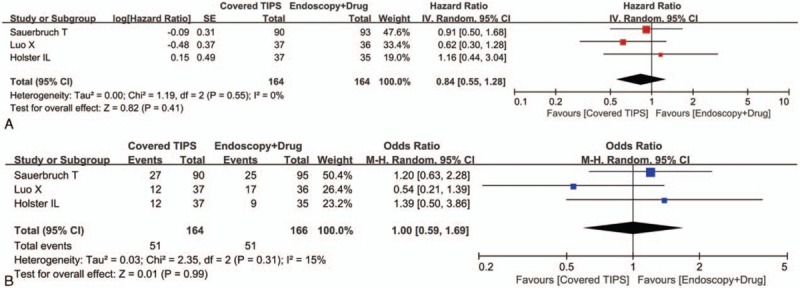
Forest plots comparing the overall survival between covered TIPS and drug plus endoscopic therapy groups. The hazard ratio for the overall survival (A) and the odds ratio for mortality (B) were calculated. TIPS = transjugular intrahepatic portosystemic shunt.

All of the 3 randomized controlled trials provided the cumulative data regarding overall survival. The meta-analysis demonstrated that the covered TIPS group had a statistically similar overall survival as compared to the drug plus endoscopic therapy group (HR = 0.84, 95% CI = 0.55–1.28, *P* = 0.41) (Fig. 2A). The heterogeneity among studies was not statistically significant (*I*^2^ = 0%, *P* = 0.55).

All of the 3 randomized controlled trials provided the overall data regarding death. The meta-analysis demonstrated that the covered TIPS group had a statistically similar risk of death as compared to the drug plus endoscopic therapy group (OR = 1.00, 95% CI = 0.59–1.69, *P* = 0.99) (Fig. 2B). The heterogeneity among studies was not statistically significant (*I*^2^ = 15%, *P* = 0.31).

### 3.4 Variceal rebleeding

**Figure 3:**
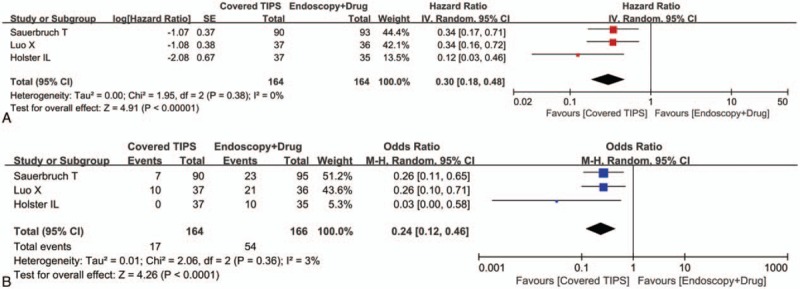
Forest plots comparing the variceal rebleeding between covered TIPS and drug plus endoscopic therapy groups. The hazard ratio for the risk of being free of variceal rebleeding (A) and the odds ratio for the risk of variceal rebleeding (B) were calculated. TIPS = transjugular intrahepatic portosystemic shunt.

All of the 3 randomized controlled trials provided the cumulative data regarding the rate of being free of variceal rebleeding. The meta-analysis demonstrated that the covered TIPS group had a significantly higher rate of being free of variceal rebleeding than the drug plus endoscopic therapy group (HR = 0.30, 95% CI = 0.18–0.48, *P* < 0.00001) (Fig. 3A). The heterogeneity among studies was not statistically significant (*I*^2^ = 0%, *P* = 0.38).

All of the 3 randomized controlled trials provided the overall data regarding variceal rebleeding. The meta-analysis demonstrated that the covered TIPS group had a significantly lower risk of variceal rebleeding than the drug plus endoscopic therapy group (OR = 0.24, 95% CI = 0.12–0.46, *P* < 0.0001) (Fig. 3B). The heterogeneity among studies was not statistically significant (*I*^2^ = 3%, *P* = 0.36).

### 3.5 Hepatic encephalopathy

**Figure 4:**
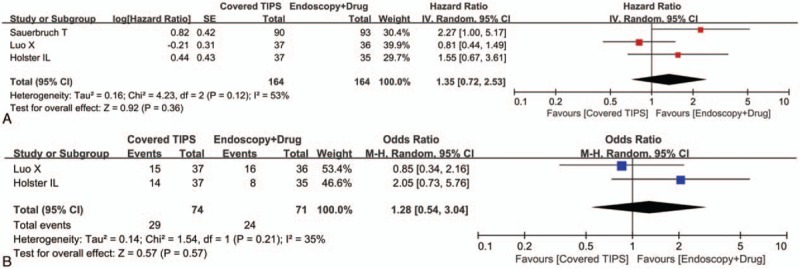
Forest plots comparing the hepatic encephalopathy between covered TIPS and drug plus endoscopic therapy groups. The hazard ratio for the risk of being free of hepatic encephalopathy (A) and the odds ratio for the risk of hepatic encephalopathy (B) were calculated. TIPS = transjugular intrahepatic portosystemic shunt.

All of the 3 randomized controlled trials provided the cumulative data regarding the rate of being free of hepatic encephalopathy. The meta-analysis demonstrated that the covered TIPS group had a statistically similar rate of being free of hepatic encephalopathy as compared to the drug plus endoscopic therapy group (HR = 1.35, 95% CI = 0.72–2.53, *P* = 0.36) (Fig. 4A). The heterogeneity among studies was statistically significant (*I*^2^ = 53%, *P* = 0.12).

Two of the 3 randomized controlled trials provided the overall data regarding hepatic encephalopathy. The meta-analysis demonstrated that the covered TIPS group had a statistically similar rate of hepatic encephalopathy as compared to the drug plus endoscopic therapy group (OR = 1.28, 95% CI = 0.54–3.04, *P* = 0.57) (Fig. 4B). The heterogeneity among studies was not statistically significant (*I*^2^ = 35%, *P* = 0.21).

## 4 Discussion

Previous meta-analyses had extensively evaluated the role of TIPS versus endoscopic treatment for the prevention of variceal rebleeding in liver cirrhosis. Before explaining our findings, their data should be fully reviewed. In 1999, Luca et al^[4]^ conducted a meta-analysis of 11 randomized controlled trials involving 750 patients and found that TIPS significantly reduced the risk of variceal rebleeding (pooled difference: –31%, [95% CI: –39% to –23%]), but significantly increased the risk of hepatic encephalopathy (pooled difference: +16%, [95% CI: +10% to +22%]) without any significant changes in death due to all causes (pooled difference: +2%, [95% CI: –4% to +9%]) or bleeding (pooled difference: –5%, [95% CI: –11% to +6%]). In the same year, Papatheodoridis et al^[5]^ also conducted a meta-analysis of 11 randomized controlled trials involving 811 patients and found that the endoscopic therapy group had a significantly higher incidence of variceal rebleeding (OR = 3.8, [95% CI: 2.8–5.2]), a similar mortality (OR = 0.97, [95% CI: 0.71–1.34]), and a significantly lower incidence of post-treatment encephalopathy (OR = 0.43, [95% CI: 0.71–1.34]) than the TIPS group. In 2002, Burroughs and Vangeli^[6]^ conducted an updated meta-analysis of 13 randomized controlled trials involving 948 patients and found that TIPS did not have any significant survival benefits (OR = 0.875, [95% CI: 0.65–1.17]), but had a significant reduction in rebleeding (OR = 3.28, [95% CI: 2.28–4.72]) and a significant increase in hepatic encephalopathy (OR = 0.48, [95% CI: 0.34–0.67]). In 2008, Zheng et al^[19]^ conducted a meta-analysis of 12 randomized controlled trials involving 883 patients and found that TIPS significantly decreased the incidence of variceal rebleeding (OR = 0.32, [95% CI: 0.24–0.43]) and deaths due to rebleeding (OR = 0.35, [95% CI: 0.18–0.67], but significantly increased the rate of post-treatment encephalopathy (OR = 2.21, [95% CI: 1.61–3.03]) and had a similar incidence of all-cause deaths (OR = 1.17, [95% CI: 0.85–1.61]). Collectively, all of them had a similar conclusion that TIPS did decrease the incidence of variceal rebleeding, but increased the incidence of hepatic encephalopathy without any survival benefits. On the basis of the fact, TIPS should be considered after drug and endoscopic therapy failed. Several characteristics of previous meta-analyses should also be emphasized. First, in the first 2 meta-analyses by Luca and Papatheodoridis,^[4,5]^ all the included randomized controlled trials were published between 1995 and 1998. In the latter 2 meta-analyses by Burroughs and Vangeli^[6]^ and Zheng et al,^[19]^ all the included randomized controlled trials were published between 1995 and 2002. Second, in nearly half of the randomized controlled trials included in the 4 previous meta-analyses, the control group was endoscopic sclerotherapy alone^[20–25]^ or in combination with propranolol.^[26]^ This was largely inconsistent with the current practice guideline recommendation that endoscopic variceal ligation, rather than sclerotherapy, should be considered. Third, only bare stents were employed for TIPS procedures in the 4 previous meta-analyses.

To the best of our knowledge, our study is the first meta-analysis regarding TIPS for the secondary prophylaxis of variceal bleeding in the era of covered stents. In line with previous meta-analyses,^[4–6,19]^ we found that covered TIPS significantly decreased the incidence of variceal rebleeding without any remarkable effect on overall survival. Contrarily, we found that covered TIPS did not significantly increase the incidence of hepatic encephalopathy. These findings might influence the future treatment algorithm for the secondary prophylaxis of variceal bleeding in liver cirrhosis.

Undoubtedly, HVPG-guided treatment is a more reasonable choice for the control group in the randomized controlled trial by Sauerbruch et al.^[18]^ In the control group, the decision to undergo endoscopic therapy after the use of nonselective beta blockers was made according to the HVPG response. In detail, the responders continued nonselective beta blockers, but the nonresponders were switched to endoscopic therapy. However, this needed to be re-considered based on the several pieces of evidence. First, in an early study by Bureau et al^[27]^, 34 cirrhotic patients who received propranolol for the prevention of first bleeding or rebleeding were evaluated by serial HVPG measurements. In the first follow-up HVPG measurement, the responders continued propranolol alone, but the nonresponders received the combination therapy of propranolol and isosorbide-5 mononitrate. In the second follow-up HVPG measurement, the responders continued drug therapy, but the nonresponders underwent endoscopic variceal ligation. They found that the hemodynamic response to drug, but not additional endoscopic variceal ligation in nonresponders, was an independent predictor for the risk of variceal bleeding. Second, in a randomized controlled trial by Garcia-Pagan et al^[28]^, 158 cirrhotic patients were assigned to nadolol plus isosorbide-5-mononitrate alone or in combination with endoscopic band ligation for the prevention of variceal rebleeding, and 135 of them had repeat HVPG measurements during follow-up. In the nonresponders, the cumulative risk of rebleeding was similar between the 2 groups. Third, in a recent study by Reiberger et al^[29]^, 104 cirrhotic patients who received propranolol for the prevention of the first variceal bleeding were evaluated by serial HVPG measurements. In the first follow-up HVPG measurement, the responders continued propranolol, but the nonresponders were switched to carvedilol. In the second follow-up HVPG measurement, the responders continued drug therapy, but the nonresponders were switched to endoscopic variceal ligation. They found that the risk of variceal rebleeding, hepatic decompensation, and mortality were significantly lower in the responders to drug therapy than the nonresponders who were switched to endoscopic variceal ligation. Taken together, additional endoscopic therapy might not be given in the nonresponders to drug therapy. Instead, in the future trial design, if TIPS was directly added in the nonresponders to drug therapy, the results would be more valuable.

Major strengths should be as follows: (1) only randomized controlled trials were included in our meta-analysis; (2) the risk of bias was relatively low in the 3 included randomized controlled trials; (3) only random-effect models were employed; (4) both HRs and ORs were calculated to confirm the effect of covered TIPS; and (5) the heterogeneity among studies was rarely observed. Several limitations should also be clarified: (1) the number of relevant randomized controlled trials was relatively small; (2) the study population was not completely homogeneous among studies; (3) the brands of covered stents for TIPS procedures were heterogeneous among studies; (4) the treatment strategy in the control group was different among studies; and (5) a randomized controlled trial by Luo et al^[17]^ was registered after the patient enrollment.

In conclusion, covered TIPS decreased the development of variceal rebleeding without any significant increase in the development of hepatic encephalopathy and with similar overall survival. Whether the use of covered stents for TIPS will revolutionize the strategy of secondary prophylaxis of variceal bleeding in liver cirrhosis should be further explored by more well-designed clinical trials.

## Supplementary Material

Supplemental Digital Content

## References

[1] Garcia-Tsao G Bosch J Management of varices and variceal hemorrhage in cirrhosis. N Engl J Med 2010;362:823–32. 20200386 10.1056/NEJMra0901512

[2] Garcia-Tsao G Sanyal AJ Grace ND Prevention and management of gastroesophageal varices and variceal hemorrhage in cirrhosis. Hepatology 2007;46:922–38. 17879356 10.1002/hep.21907

[3] de Franchis R Expanding consensus in portal hypertension: Report of the Baveno VI Consensus Workshop: stratifying risk and individualizing care for portal hypertension. J Hepatol 2015;63:743–52. 26047908 10.1016/j.jhep.2015.05.022

[4] Luca A D’Amico G La Galla R TIPS for prevention of recurrent bleeding in patients with cirrhosis: meta-analysis of randomized clinical trials. Radiology 1999;212:411–21. 10429698 10.1148/radiology.212.2.r99au46411

[5] Papatheodoridis GV Goulis J Leandro G Transjugular intrahepatic portosystemic shunt compared with endoscopic treatment for prevention of variceal rebleeding: a meta-analysis. Hepatology 1999;30:612–22. 10462365 10.1002/hep.510300316

[6] Burroughs AK Vangeli M Transjugular intrahepatic portosystemic shunt versus endoscopic therapy: randomized trials for secondary prophylaxis of variceal bleeding: an updated meta-analysis. Scand J Gastroenterol 2002;37:249–52. 11916185 10.1080/003655202317284138

[7] Bureau C Garcia-Pagan JC Otal P Improved clinical outcome using polytetrafluoroethylene-coated stents for TIPS: results of a randomized study. Gastroenterology 2004;126:469–75. 14762784 10.1053/j.gastro.2003.11.016

[8] Bureau C Pagan JC Layrargues GP Patency of stents covered with polytetrafluoroethylene in patients treated by transjugular intrahepatic portosystemic shunts: long-term results of a randomized multicentre study. Liver Int 2007;27:742–7. 17617116 10.1111/j.1478-3231.2007.01522.x

[9] Perarnau JM Le Gouge A Nicolas C Covered vs. uncovered stents for transjugular intrahepatic portosystemic shunt: a randomized controlled trial. J Hepatol 2014;60:962–8. 24480619 10.1016/j.jhep.2014.01.015

[10] Yang Z Han G Wu Q Patency and clinical outcomes of transjugular intrahepatic portosystemic shunt with polytetrafluoroethylene-covered stents versus bare stents: a meta-analysis. J Gastroenterol Hepatol 2010;25:1718–25. 21039832 10.1111/j.1440-1746.2010.06400.x

[11] Qi X Guo X Fan D A trend toward the improvement of survival after TIPS by the use of covered stents: a meta-analysis of two randomized controlled trials. Cardiovasc Intervent Radiol 2015;38:1363–4. 25274207 10.1007/s00270-014-0996-9

[12] Qi X Jia J Bai M Transjugular intrahepatic portosystemic shunt for acute variceal bleeding: a meta-analysis. J Clin Gastroenterol 2015;49:495–505. 25127113 10.1097/MCG.0000000000000205

[13] Qi X Liu L Wang D Hepatic resection alone versus in combination with pre- and post-operative transarterial chemoembolization for the treatment of hepatocellular carcinoma: a systematic review and meta-analysis. Oncotarget 2015;6:36838–59. 26451613 10.18632/oncotarget.5426 PMC4742214

[14] Qi X Tang Y An D Radiofrequency ablation versus hepatic resection for small hepatocellular carcinoma: a meta-analysis of randomized controlled trials. J Clin Gastroenterol 2014;48:450–7. 24172183 10.1097/MCG.0000000000000008

[15] Qi X Wang D Su C Hepatic resection versus transarterial chemoembolization for the initial treatment of hepatocellular carcinoma: a systematic review and meta-analysis. Oncotarget 2015;6:18715–33. 26243835 10.18632/oncotarget.4134 PMC4621923

[16] Holster IL Tjwa ET Moelker A Covered transjugular intrahepatic portosystemic shunt versus endoscopic therapy + beta-blocker for prevention of variceal rebleeding. Hepatology 2016;63:581–9. 26517576 10.1002/hep.28318

[17] Luo X Wang Z Tsauo J Advanced cirrhosis combined with portal vein thrombosis: a randomized trial of TIPS versus endoscopic band ligation plus propranolol for the prevention of recurrent esophageal variceal bleeding. Radiology 2015;276:286–93. 25759969 10.1148/radiol.15141252

[18] Sauerbruch T Mengel M Dollinger M Prevention of rebleeding from esophageal varices in patients with cirrhosis receiving small-diameter stents versus hemodynamically controlled medical therapy. Gastroenterology 2015;149:660–8. e1. 25989386 10.1053/j.gastro.2015.05.011

[19] Zheng M Chen Y Bai J Transjugular intrahepatic portosystemic shunt versus endoscopic therapy in the secondary prophylaxis of variceal rebleeding in cirrhotic patients: meta-analysis update. J Clin Gastroenterol 2008;42:507–16. 18344888 10.1097/MCG.0b013e31815576e6

[20] Cabrera J Maynar M Granados R Transjugular intrahepatic portosystemic shunt versus sclerotherapy in the elective treatment of variceal hemorrhage. Gastroenterology 1996;110:832–9. 8608893 10.1053/gast.1996.v110.pm8608893

[21] Cello JP Ring EJ Olcott EW Endoscopic sclerotherapy compared with percutaneous transjugular intrahepatic portosystemic shunt after initial sclerotherapy in patients with acute variceal hemorrhage. A randomized, controlled trial. Ann Intern Med 1997;126:858–65. 9163286 10.7326/0003-4819-126-11-199706010-00002

[22] Sanyal AJ Freedman AM Luketic VA Transjugular intrahepatic portosystemic shunts compared with endoscopic sclerotherapy for the prevention of recurrent variceal hemorrhage. A randomized, controlled trial. Ann Intern Med 1997;126:849–57. 9163285 10.7326/0003-4819-126-11-199706010-00001

[23] Merli M Salerno F Riggio O Transjugular intrahepatic portosystemic shunt versus endoscopic sclerotherapy for the prevention of variceal bleeding in cirrhosis: a randomized multicenter trial. Gruppo Italiano Studio TIPS (G.I.S.T). Hepatology 1998;27:48–53. 9425916 10.1002/hep.510270109

[24] Garcia-Villarreal L Martinez-Lagares F Sierra A Transjugular intrahepatic portosystemic shunt versus endoscopic sclerotherapy for the prevention of variceal rebleeding after recent variceal hemorrhage. Hepatology 1999;29:27–32. 9862845 10.1002/hep.510290125

[25] Narahara Y Kanazawa H Kawamata H A randomized clinical trial comparing transjugular intrahepatic portosystemic shunt with endoscopic sclerotherapy in the long-term management of patients with cirrhosis after recent variceal hemorrhage. Hepatol Res 2001;21:189–98. 11673103 10.1016/s1386-6346(01)00104-8

[26] Sauer P Theilmann L Stremmel W Transjugular intrahepatic portosystemic stent shunt versus sclerotherapy plus propranolol for variceal rebleeding. Gastroenterology 1997;113:1623–31. 9352865 10.1053/gast.1997.v113.pm9352865

[27] Bureau C Peron JM Alric L A La Carte” treatment of portal hypertension: adapting medical therapy to hemodynamic response for the prevention of bleeding. Hepatology 2002;36:1361–6. 12447860 10.1053/jhep.2002.36945

[28] Garcia-Pagan JC Villanueva C Albillos A Nadolol plus isosorbide mononitrate alone or associated with band ligation in the prevention of recurrent bleeding: a multicentre randomised controlled trial. Gut 2009;58:1144–50. 19218249 10.1136/gut.2008.171207

[29] Reiberger T Ulbrich G Ferlitsch A Carvedilol for primary prophylaxis of variceal bleeding in cirrhotic patients with haemodynamic non-response to propranolol. Gut 2013;62:1634–41. 23250049 10.1136/gutjnl-2012-304038

