# Supplementary material for: Covered TIPS for secondary prophylaxis of variceal bleeding in liver cirrhosis: A systematic review and meta-analysis of randomized controlled trials
Source: Medicine (Baltimore). 2016 Dec 16;95(50):e5680. doi: 10.1097/MD.0000000000005680 (PMC5268064; doi:10.1097/MD.0000000000005680)
Supplement: Supplemental Digital Content [file md-95-e5680-s001.doc]

| **Supplementary Table 1. Risk of bias for the study by Holster** | | |
| --- | --- | --- |
| **Entry** | **Judgment** | **Support for judgment** |
| Random sequence generation (selection bias) | Low risk. | Quote:  "The randomization sequence was computer generated with the use of a concealed block size of four, stratified by Child-Pugh class." Comment: Using a computer random number generator. |
| Allocation concealment (selection bias) | Low risk. | Quote: "Patients were randomly assigned through a permanently available central telephone system to receive further endoscopic therapy in combination with b-blocker therapy (standard of care) or TIPS placement." Comment: Central allocation. |
| Blinding of participants and personnel (performance bias) | High risk. | Comment: No information provided. But the blinding of participants and personnel was not practical, because the treatment was very different between the two groups. |
| Blinding of outcome assessment (detection bias) | Unclear risk. | Comment: No information provided. |
| Incomplete outcome data addressed (attrition bias) | Low risk. | Quote: "Six patients (8%; 4 in the endoscopy1b-blocker arm, 2 in the TIPS arm) were lost-to-follow-up after a median of 22.5 months (IQR, 4.2-34.3)." Comment: Missing outcome data balanced in numbers across intervention groups, with similar reasons for missing data across groups. |
| Selective reporting (reporting bias) | Low risk. | Comments: All endpoints were reported as planned. Review authors do not believe that the reporting bias will be introduced. |

| **Supplementary Table 2. Risk of bias for the study by Luo** | | |
| --- | --- | --- |
| **Entry** | **Judgment** | **Support for judgment** |
| Random sequence generation (selection bias) | Low risk. | Quote:  "Eligible patients were assigned randomly to the TIPS or EBL group by using consecutive numbers generated by computer allocated random-digit numbers." Comment: Using a computer random number generator. |
| Allocation concealment (selection bias) | Unclear risk. | Comment: No information provided. |
| Blinding of participants and personnel (performance bias) | High risk. | Comment: No information provided. But the blinding of participants and personnel was not practical, because the treatment was very different between the two groups. |
| Blinding of outcome assessment (detection bias) | Unclear risk. | Comment: No information provided. |
| Incomplete outcome data addressed (attrition bias) | Low risk. | Quote: "Three patients (one in the TIPS group and two in the EBL group) were lost to follow-up after a mean of 7 months." Comment: Missing outcome data balanced in numbers across intervention groups, with similar reasons for missing data across groups. |
| Selective reporting (reporting bias) | Low risk. | Comments: All endpoints were reported as planned. Review authors do not believe that the reporting bias will be introduced. |

| **Supplementary Table 3. Risk of bias for the study by Sauerbruch** | | |
| --- | --- | --- |
| **Entry** | **Judgment** | **Support for judgment** |
| Random sequence generation (selection bias) | Low risk. | Quote:  "After verification of inclusion and exclusion criteria the patients were assigned (1:1) into group A or group B by fax randomization following a block randomization scheme stratifying for centers and time after index bleed (strata)." "The scheme was generated at the Institut für Medizinische Biometrie, Informatik und Epidemiologie, University of Bonn." |
| Allocation concealment (selection bias) | Low risk. | Quote:  "For allocation concealment, assignment to the respective group was performed by independent uninvolved individuals." |
| Blinding of participants and personnel (performance bias) | High risk. | Comment: No information provided. But the blinding of participants and personnel was not practical, because the treatment was very different between the two groups. |
| Blinding of outcome assessment (detection bias) | Unclear risk. | Comment: No information provided. |
| Incomplete outcome data addressed (attrition bias) | Unclear risk. | Comment: Insufficient reporting of attrition/exclusions. |
| Selective reporting (reporting bias) | Low risk. | Comments: All endpoints were reported as planned. Review authors do not believe that the reporting bias will be introduced. |
